# Barriers and facilitators to implementing evidence-based interventions in the context of a randomized clinical trial in the United States: a qualitative study

**DOI:** 10.1186/s12913-023-09079-2

**Published:** 2023-01-26

**Authors:** Elise Trott Jaramillo, Cathleen E Willging, Lisa Saldana, Shannon Self-Brown, Erin A. Weeks, Daniel J. Whitaker

**Affiliations:** 1 Pacific Institute for Research and Evaluation, 851 University Blvd. SE, Suite 101, Albuquerque, NM 87106 USA; 2 Oregon Social Learning Center, 10 Shelton McMurphey Blvd, Eugene, OR 97401 USA; 3 School of Public Health, Georgia State University, 14 Marietta St. NW, Suite 232, Atlanta, GA 30303 USA

**Keywords:** Evidence-based interventions, Randomized controlled trials, Leadership, Implementation, Qualitative research

## Abstract

**Background:**

Evidence-based interventions, which are typically supported by data from randomized controlled trials (RCTs), are highly valued by providers of human services like child welfare. However, implementing such interventions in the context of a randomized clinical trial is a complex process, as conducting an RCT adds extra tasks for providers and complicating factors for provider organizations. Utilizing the Exploration, Preparation, Implementation, and Sustainment Framework, this study examines factors that facilitate or impede success in the implementation of evidence-based interventions in the context of a largescale trial of SafeCare,® a child maltreatment intervention.

**Methods:**

Qualitative data were obtained as part of a larger mixed-methods study involving a cluster randomized trial comparing SafeCare to usual services for caregivers within nine child welfare agencies across four states. Between May and October 2017, individual interviews were conducted with a purposive sample of 21 child welfare administrators and 24 supervisors, and 19 focus groups were conducted with 84 providers. Data were coded iteratively and grouped into themes.

**Results:**

Several interconnected themes centered on facilitators and barriers to SafeCare implementation in the context of a randomized clinical trial. Facilitators included: (1) Benefits afforded through RCT participation; (2) Shared vision and sustained buy-in across system and organizational levels; and (3) Ongoing leadership support for SafeCare and the RCT. Barriers that hindered SafeCare were: (1) Insufficient preparation to incorporate SafeCare into services; (2) Perceived lack of fit, leading to mixed support for SafeCare and the RCT; and (3) Requirements of RCT participation at the provider level.

**Conclusions:**

These data yield insight into an array of stakeholder perspectives on the experience of implementing a new intervention in the context of a largescale trial. This research also sheds light on how the dynamics of conducting an RCT may affect efforts to implement interventions in complex and high-pressure contexts. Findings highlight the importance of aligning knowledge and expectations among researchers, administrators of organizations, and supervisors and providers. Researchers should work to alleviate the burdens of study involvement and promote buy-in among frontline staff not only for the program but also for the research itself.

## Background

Providers of human services, such as child welfare, place a high value on evidence-based interventions (EBIs), meaning services and treatments that have a proven research base of effectiveness. For an intervention to receive EBI status, it must be supported by evidence [1–3]. Although this evidence can come from a variety of study designs, it has conventionally come from randomized controlled trials (RCTs), a type of research design that randomly divides participants into two groups—one that receives an intervention (the experimental group) and one that does not (the control group)—and then compares outcomes between the groups [4–6]. However, embedding even the most rigorously supported EBIs in service systems is a complex process for the organizations and individuals tasked with delivering them to clients in diverse contexts [7–9]. Implementation involves balancing pressures and resources within the inner context of implementing organizations (e.g., organizational culture, scopes of work, and leadership support) and individual practitioners (e.g., openness to innovation, readiness for change, caseloads, perceptions of client needs) with those coming from the outer context of the broader system within which organizations operate (e.g., funding, procurement and contracting processes, government mandates). Moreover, much of the work of instantiating a new EBI falls on the shoulders of already busy, overworked professionals. Conducting an RCT in this context adds extra tasks for providers, such as assisting with recruitment and documenting activities in specific ways. An RCT may also introduce potentially complicating factors for provider organizations, such as how to organize and supervise providers who are randomized to different conditions. This study elucidates factors that facilitate or impede success in the implementation of EBIs in the context of an RCT by examining the experiences of child welfare professionals involved in a largescale trial of SafeCare,® a highly structured home-based behavioral skills training and education EBI to reduce and prevent child maltreatment for parents of children aged 0–5 [10–12].

SafeCare is typically delivered in homes to caregivers and parents identified as at-risk, or reported, for child maltreatment, to improve parenting knowledge and skills and to create safer living environments for children. While the majority of child welfare interventions focus on preventing child abuse through cultivating positive parent–child relationships and non-coercive forms of discipline, SafeCare is one of the only EBIs that promotes skills to prevent child neglect, which is the most common reason that caregivers are referred to child welfare [13]. SafeCare is also among the few interventions that are suitable for very young children (i.e., ages 0–5) who are most vulnerable to neglect. The intervention consists of three structured modules focused on child health, home safety, and parent–child or parent-infant interaction. Previous studies have demonstrated that SafeCare is effective in increasing parenting skills and reducing child maltreatment recidivism [12, 14–16].

The implementation of EBIs is a dynamic, multi-stage process. The Exploration, Preparation, Implementation, and Sustainment (EPIS) model conceptualizes the implementation process as occurring in four stages: Exploration (consideration of an EBI), Preparation (planning to carry out the EBI), Implementation (training and provision of the EBI), and Sustainment (maintaining the EBI with fidelity) [7, 17]. Within each phase, implementation is influenced by a multitude of inner- and outer-context factors. Key inner-context factors include provider attitudes toward EBIs, perceived appropriateness and fit of the new EBI, leadership support, and organizational climate and culture surrounding the adoption of new practices. Outer-context factors may include funding arrangements and policies affecting child welfare. For this study, the EPIS model illuminates key commonalities and differences across diverse service settings that affect implementation of an EBI from the perspectives of child welfare professionals responsible for in-home parenting services (e.g., program administrators, supervisors, and frontline staff). The purpose of this study is to yield insight into an array of stakeholder perspectives on the experience of implementing a new intervention in the context of a largescale RCT. This research also sheds light on how the dynamics of conducting an RCT may affect efforts to implement interventions in complex and high-pressure contexts.

## Methods

Qualitative data were obtained as part of a larger mixed-methods study involving a cluster randomized trial comparing SafeCare to usual services for caregivers within several United States child welfare agencies across four states [12]. Qualitative research is useful for understanding the perceptions and experiences of people involved in research or who are otherwise affected by it. Such approaches are designed to yield insight into service delivery contexts and the on-the-ground dynamics of research and implementation processes, in addition to the intended and unintended consequences associated with these processes. For the larger study, data from the clinical trial examining the effects of SafeCare on a variety of parenting-related outcomes were complemented by qualitative interviews with child welfare professionals (e.g., administrators, supervisors, and providers) and parents about their perspectives on SafeCare and usual services, focusing on inner- and outer-context factors affecting implementation, reactions, and perceived impacts associated with in-home parenting services. The project was guided by an advisory board that included service users. The current analysis focuses on the perspectives of child welfare professionals to illuminate the factors that supported and hindered the implementation of SafeCare within child welfare agencies.

### Study setting

This study took place in nine child welfare agencies based in four of the United States. This included two county-based child welfare departments in one state, and seven community-based organizations (CBOs) across the remaining three states. These agencies served families who had been referred by their local child protective service system due to reports of possible abuse and neglect, which may or may not have been substantiated, or risk factors for abuse and neglect.

### Participation in the RCT

As part of the RCT, existing teams of providers in each agency (e.g., teams serving distinct geographic areas) were randomly assigned to an experimental or control condition. Teams in the experimental condition received SafeCare training and were asked to provide SafeCare to parents, which involved conducting 18–20 weekly sessions. Teams in the control condition continued providing usual services that mainly consisted of support, parenting education, and case management activities. Usual services involved meeting with parents weekly or as needed.

All providers (SafeCare and control) were asked to assist with the recruitment of families into the trial. They were trained to introduce the study to each eligible family (i.e., with a child aged 0–5 who was the target of services), ask caregivers if they might be interested in participating, and if so, to provide their name and contact information to the research team. Providers were to document basic non-identified information about caregivers who declined to participate. Providers in the experimental condition were also expected to comply with all the requirements of SafeCare implementation set by the National Safecare Training and Research Center (NSTRC), which included fidelity assessments with feedback by certified coaches and ongoing team meetings. This entailed recording sessions with caregivers for quality assurance and using a smartphone app to log clinical assessments that are part of the SafeCare model, as well as basic demographic information about each caregiver, caregiver engagement at each session, and caregiver satisfaction with services. Providers in the control condition were asked to use the app to record their session activities, including type of services provided and the approximate number of minutes spent. All provider teams met regularly with the research team to discuss recruitment progress.

### Implementation study

In addition to assessing parenting outcomes for families receiving SafeCare compared to usual services, the parent study also examined the implementation process via qualitative interviews with providers and their supervisors, which are the focus of the present analysis. The degree to which SafeCare was successfully implemented in each of the nine sites was assessed using the Stages of Implementation Completion (SIC), an eight-stage tool that measures implementation process and milestones across three phases: pre-implementation, implementation, and sustainment [18, 19]. The SIC was adapted for the SafeCare implementation process using the standard SIC adaptation process [20]. The research team tracked each study site through the eight SIC stages, from Engagement through Competency, by recording the date that implementation activities were completed by a site. Sites were scored using standard SIC scores including: (1) duration (i.e., time taken for completion of activities), (2) proportion (i.e., percentage of activities completed), and (3) final stage (i.e., the furthest point in the implementation process reached). Based on their scores, sites were then classified as either “high” (i.e., having completed the full implementation process through the Competency stage) or “low” (i.e., having discontinued implementation before achieving the Competency stage). Of the sites in this analysis, five were classified as high implementation sites and four were classified as low implementation sites.

### Participants

Purposive sample selection in qualitative research is designed to represent the breadth of views related to study issues, focusing on people who can discuss the most relevant issues under investigation [21]. Participants in the qualitative component of the implementation study included 84 providers employed by the child welfare departments and organizations that participated in the project, who were tasked with delivering services to families (including 43 in the experimental condition [i.e., delivering SafeCare] and 41 in the control condition [i.e., delivering usual services]); 23 supervisors, who provided clinical support to providers and helped oversee their cases, and 21 administrators of child welfare agencies and organizations. Table 1 summarizes participant characteristics among the child welfare professionals. Participants received $50 for their time and knowledge shared. All participants also signed a written informed consent document. To protect their anonymity, names and identifying features of the participants and their workplaces have been removed from the data reported here. All processes were approved by the Georgia State University Institutional Review Board.

**Table 1:** Demographic Characteristics of Child Welfare Professional Participants^a^

	*SafeCare Providers* *N* = *43*	*Usual Services Providers* *N* = *41*	*Administrators* *N* = *21*	*SafeCare* *Supervisors* *N* = *11*	*Usual Services Supervisors* *N* = *13*
	*N(%) or M(*± *SD)*	*N(%) or M(*± *SD)*	*N(%) or M(*± *SD)*	*N(%) or M(*± *SD)*	*N(%) or M(*± *SD)*
Gender					
Male Female	4 (10.5%) 34 (89.5%)	5 (12.2%) 36 (87.8%)	5 (23.8%) 16 (76.2%)	2 (18.2%) 9 (81.8%)	0 (0%) 13 (100.0%)
Age at Interview	40.3(± 12.4)	35(± 11.8)	49.7(± 10.3)	43.2(± 10.1)	38.0(± 5.1)
Education					
High School Graduate Some College College Degree Some Graduate School Master’s Degree	2 (5.3%) 4 (10.5%) 22 (57.9%) 1 (2.6%) 9 (23.7%)	0 (0%) 0 (0%) 30 (75.0%) 1 (2.5%) 9 (22.5%)	0 (0%) 0 (0%) 3 (16.7%) 2 (11.1%) 13 (72.2%)	0 (0%) 1 (9.1%) 7 (63.6%) 1 (9.1%) 2 (18.2%)	0 (0%) 0 (0%) 5 (41.7%) 1 (8.3%) 6 (50.0%)
Race					
Caucasian African American Native American Asian/Pacific Islander Other	27 (71.1%) 10 (26.3%) 0 (0%) 0 (0%) 1 (2.7%)	30 (73.2%) 7 (17.1%) 2 (4.9%) 1 (2.4%) 1 (2.4%)	18 (90.0%) 2 (10.0%) 0 (0%) 0 (0%) 0 (0%)	10 (90.9%) 0 (0%) 0 (0%) 0 (0%) 1 (9.1%)	11 (84.6%) 1 (7.7%) 0 (0%) 1 (7.7%) 0 (0%)
Hispanic	3 (7.9%)	4 (9.8%)	0 (0%)	1 (9.1%)	1 (7.7%)
Tenure at Current Agency	5.5(± 6.4)	3.3(± 4.1)	11.7(± 8.3)	7.1(± 5.2)	7.8(± 4.3)

^a^Some participants elected not to provide demographic data

### Data collection

Twenty-one interviews were conducted with child welfare administrators and 24 were conducted with supervisors. Nine interviews were conducted by phone due to the lack of availability of the interviewee when the interviewers were on site. Nineteen focus groups (averaging 3–5 participants each) were conducted with providers to encourage discussion of multiple perspectives and maximize participation of providers. These occurred over a 6-month period between May and October 2017. Interviews and focus groups were conducted by anthropologists with advanced degrees. Individual interviews lasted approximately 45 to 60 min, and focus groups lasted approximately 60 to 90 min. All interviews and focus groups were digitally recorded and professionally transcribed.

The interview and focus group guides consisted of open-ended questions that were tailored to each type of participant. A single guide was developed for use among all administrators, which touched on SafeCare and usual services. The guides for supervisors and providers were further distinguished by experimental (SafeCare) or control conditions (usual services). Questions focused on professionals’ roles and responsibilities; knowledge about and experience with EBIs and the larger RCT; and factors affecting the implementation of SafeCare and the RCT, including organizational and leadership support, staffing and caseloads, and client responses. The guides are described in Tables 2 and 3.

**Table 2:** Overview of Interview Guide for Child Welfare Department and CBO Administrators

• Roles and responsibilities
• Families served by organization
• Organization’s experience with EBIs
• Exposure to, knowledge about, and experience with SafeCare
• Organizational interest in, and expectations related to, SafeCare implementation
• Organizational/individual factors affecting agency capacity to implement and sustain SafeCare
• Home visitor and supervisor buy-in and support for SafeCare implementation
• Leadership support for SafeCare implementation
• Staffing needs related to SafeCare implementation
• Client response to SafeCare implementation

**Table 3:** Overview of Interview/Focus Group Guides for Supervisors and Providers

Experimental Condition (SafeCare)	Control Condition (Usual Services)
• Roles and responsibilities • Families served by organization • Experience with EBIs • Exposure to, knowledge about, and experience with SafeCare • Expectations related to SafeCare implementation, including caseloads • Training and coaching in SafeCare model • Organizational factors affecting staff capacity to implement SafeCare • Leadership support for SafeCare implementation • Home visitor response to SafeCare • Client response to SafeCare implementation	• Roles and responsibilities • Families served by organization • Experience with EBIs • Organizational factors affecting staff capacity to provide services to clients • Perceptions of caseloads • Perceptions and knowledge of SafeCare • Staffing needs to support EBIs • Client responses to services • Leadership and supervisor support • Study participation and randomization

### Data analysis

Four anthropologists with advanced degrees took part in the analytic process. Two team members applied an iterative process to review and analyze the transcripts using Dedoose VERSION 7.6.17, a web-based qualitative data management and analysis application. They began by assigning codes to segments of text ranging from a phrase to several paragraphs based a priori on topic areas and questions making up the interview guides [21]. Codes derived from key sensitizing concepts from the EPIS framework, including the constructs of outer and inner contexts, and the broader implementation literature (e.g., leadership, appropriateness, and adoption), were intended to provide a general sense of reference for our analysis, and enable us to analyze the salience and meaning of these concepts for participants based on their own reflections on their perceptions and experiences [21]. Focused coding was then used to determine which concepts or themes emerged frequently and which represented unusual or particularly important issues to the participants. Two team members independently coded sets of transcripts, creating a codebook to guide this process and detailed memos that described and linked codes to each theme. The results of this work were further reviewed by the remaining two members of the analytic team, who also participated in the process of comparing the codes with one another and grouping together those with similar content or meaning into broad themes [22]. Discrepancies in coding and analysis were identified during this process and resolved during team meetings.

## Results

Several interconnected themes centered on facilitators and barriers to SafeCare implementation in an RCT context. Facilitators included: (1) Benefits afforded through RCT participation; (2) Shared vision and sustained buy-in across system and organizational levels; and (3) Ongoing leadership support for SafeCare and the RCT. Barriers that hindered SafeCare—especially at low implementation sites—were: (1) Insufficient preparation to incorporate SafeCare into services; (2) Perceived lack of fit, leading to mixed support for SafeCare and the RCT; and (3) Requirements of RCT participation at the provider level.

### Facilitators to SafeCare implementation in an RCT context

#### Benefits afforded through RCT participation

The decision to implement SafeCare at most sites was reportedly predicated in part on perceptions among organizational leaders that EBIs were desirable to increase the competitiveness of child welfare providers and to keep up with dominant trends. Several administrators and supervisors agreed that decisions to incorporate EBIs into service portfolios were based on the belief that proficiency in such programs would increase their likelihood of procuring future contracts and funding. One system-level administrator explained that, “We felt that this [SafeCare] would be something that could be set up to be in our next set of contracts and so we wanted to make sure we were on the cutting edge.” In addition, child welfare professionals also reported pressures from the judicial system to use EBIs and noted that parents were also attracted by the prospect of taking part in a program with proven results. One SafeCare provider observed, “Everybody, not just for the courts but like the families you work with too they’re like, ‘Oh if it’s evidence-based, studies show... [it works].'"

Another motivation for several sites to take part in an RCT to implement SafeCare was the opportunity to document the outcomes of services they provided to families, such as improvements in recidivism for child maltreatment, family reunification, parenting skills, relationships, and safe home environments. For example, one provider noted, “After a year... we’ve had several reunifications because parents have completed SafeCare that I think if they wouldn’t have they wouldn’t have been to that point.” In addition, participation in the RCT afforded sites access to training and coaching in an EBI that normally would have to be purchased at considerable expense. However, while these forms of support from the RCT were reportedly very valuable in the high implementation sites, those benefits were not realized at sites where implementation did not progress to the point where coaching and training could occur.

#### Shared vision and sustained buy-in across system and organizational levels

In the sites classified as “high” implementation sites, both system and organizational leaders were described as actively championing SafeCare beginning in the EPIS-defined Preparation phase and continuing throughout the Implementation phase. A single government child welfare administrator who had positive experiences with SafeCare in another state eagerly introduced it to CBO leaders at these specific sites, urging them to apply to take part in the RCT. Rather than presenting use of SafeCare as a mandate, they^1^ “gave [CBO leaders] support and encouragement around applying for it.” The CBO leaders, in turn, were characterized as espousing an organizational culture of “trying new things,” as described by one organizational administrator: “Everybody was on board to try something.... We have to try new things and see what works, we believe in evidence-based practice.... They [CBO leaders] were all about trying it and I was really surprised by the enthusiasm and work ethic.”

The administrators at high implementation sites emphasized that efforts to elicit buy-in for a new intervention take time and require shared vision across both system and organizational leaders. A CBO administrator noted that the CEO of their agency had communicated with state child welfare professionals about the SafeCare curriculum and was aware that they also wanted to implement SafeCare. In contrast, professionals at low implementation sites repeatedly referred to a lack of buy-in at all levels. In multiple sites, insufficient buy-in among staff was attributed to a delay between training and implementation that lasted many months. A supervisor at one such site commented, “That gap hurt us because people had all this energy at the beginning and it sort of fizzled out because they didn’t take that momentum and go with it.” In another low implementation site, an administrator rated staff enthusiasm for the intervention as low, noting a loss of buy-in for SafeCare following substantial staff turnover. In other struggling sites, the lack of buy-in was attributed to staff skepticism regarding the intervention itself. One system-level administrator commented, “The biggest barrier was that our workers were not sold on SafeCare as a good, useful intervention that would save workers time and lead to better outcomes for their families.” This individual observed that supervisors had not bought in, and had influenced their staff not to try too hard to make SafeCare work: “I heard that some people were very much not engaged in the training, like checking voicemail and email, and even maybe badmouthing it, whispering to their colleagues like, ‘Oh, we’ll never do this,’ or ‘This will never work.’” This perception among staff and the consequent lack of buy-in for the intervention and the research was likely due to several other barriers, described in following sections.

#### Ongoing leadership support for SafeCare and the RCT

In addition to having leaders who enthusiastically championed the use of SafeCare, participants at the high implementation sites reported that they had benefited from their leaders actively encouraging the intervention and providing support throughout all phases of implementation. In one site, a SafeCare supervisor appreciated that leaders attended monthly meetings, provided direct supervision, and wanted the organization to progress to the point of having in-house SafeCare trainers. A second supervisor commented, “Leadership did a good job of really having a lot of conversations about it [SafeCare implementation].” A third supervisor stressed the influence of leadership in securing staff support: “It’s administration and the supervisor level of just being really excited about the program and selling it to our staff.”

Supportive leadership involved proactive communication across levels of staff. A supervisor at one high implementation site commented, “[This agency] is really good at when they have the information they share it. Like our staff are never left in the dark about stuff.” At another successful site, an administrator described how CBO leaders sought to understand the experiences of frontline staff to better support them: “We have an “all in this together” approach. It was important to us that when we did SafeCare [that] we did that same approach so that from the top-down we understood what we were asking our staff to undertake.” This individual explained that organizational administrators insisted that supervision staff be trained in SafeCare so that they could best support their teams, even if they did not deliver SafeCare themselves.

In contrast, although leaders at the low implementation sites reported favorable perceptions of SafeCare, support for the intervention was not experienced consistently by staff. At one site, administrators wanted staff to focus on traditional clinical work and complained that SafeCare was interfering with the agency’s ability to offer other vital services to families. Turnover of leadership also presented challenges to SafeCare implementation and sustainment in low sites, especially when champions at both system and organizational levels vacated their positions, leaving leaders at the helm who knew little about SafeCare.

Leaders at the low implementation sites also did not involve themselves in solving the day-to-day challenges of implementation. Providers at several of these sites intimated that organizational leaders neither foresaw, nor addressed, issues that threatened implementation: “It’s just a disconnect with each level, with us being workers, and our supervisors and the administration.... They think it could work, obviously. They also are not the ones in the field... [T]here’s a lot of bumps that we hit, a lot of potholes that we’re encountering, and they’re not trying to understand those roadblocks that we’re having with our clientele.” These providers did not view their leaders as understanding the realities of implementation, suggesting that they were therefore unable to offer pragmatic solutions to staff struggling with the practicalities of delivering SafeCare to families with diverse needs, or with incorporating tasks associated with SafeCare and the research study into their daily workloads.

### Barriers to SafeCare implementation in an RCT context

#### Insufficient preparation to incorporate SafeCare into services

A significant barrier to SafeCare implementation identified by the child welfare professionals in low implementation sites was a lack of planning during the EPIS-defined Preparation and early Implementation phases to incorporate SafeCare into the existing work of the organizations and staff. Several professionals at all levels voiced regrets that they had not prepared sufficiently for the added complexities of implementing a new intervention, suggesting that they should have spent more time asking questions and enhancing readiness. For some sites, contracts with government child welfare departments did not include specifications regarding SafeCare, while standard policies and procedures also remained unchanged. Deficient planning created a dearth of guidance for supervisors and providers in their efforts to use SafeCare. An administrator at a low implementation site recognized the need to better establish SafeCare in system and organizational contexts, saying, “If we’re going to do this, we need to put it in policy and make it part of the services that we provide."

The lack of sufficient integration of SafeCare into contracts, policies, and procedures during the Preparation phase created confusion around how to integrate SafeCare into the existing caseloads of providers. At one site, administrators hired additional staff and initiated changes to the way providers were assigned cases, after discovering that SafeCare cases were more labor-intensive than others. An administrator at this site acknowledged that the usual process for determining both caseloads and financial remuneration for staff were not suited for SafeCare, explaining, “How we pay our staff is based on number of cases and so if you had SafeCare in every case, maybe with the complexity of cases and interactions that cases had to have, sometimes they’re not able to have as many.” A supervisor elsewhere stated, “We’re always understaffed so we have to overburden them [providers] to meet our contractual number with the state.” Others said that providers had so many cases on their dockets that SafeCare was not a priority.

For their part, providers expressed reservations about the feasibility of incorporating SafeCare into their existing workloads, based in part on the belief that SafeCare required extra time and effort to deliver with fidelity. At several sites, where providers juggled multiple competing demands, complaints that staff were unable to dedicate time to implement SafeCare were pervasive. One provider explained, “We’re all here doing a fulltime job of managing cases. SafeCare in itself can be a fulltime job.”

A related challenge stemming from a lack of preparation for the implementation of SafeCare was a common lack of confidence among providers and supervisors about how to optimally deploy SafeCare to meet the goals and needs of their clients. For example, a SafeCare supervisor reflected, “We were really excited to get this [SafeCare] started but I don’t think we really sat down and thought about: ‘Are our families appropriate?’... I just never really thought about the [SafeCare] modules and how are we going to incorporate this with our families.” Some supervisors were not convinced that providers should assume responsibility for implementing a parenting program when they should instead be “operating more as case managers and really working with families to help connect them to services."

#### Perceived lack of fit, leading to mixed support for SafeCare and the RCT

The majority of participants in this study expressed that they had entered the early EPIS-defined Implementation phase with positive perceptions of SafeCare in theory, while simultaneously doubting the feasibility of successfully utilizing the intervention. One provider in the control condition harkened to prior experiences with failed implementation of promising programs, expressing skepticism that SafeCare would be permanently integrated into service portfolios: “The difficulty for me is I’ve been around long enough that [I’ve seen] a lot of these things come in and they’re these wonderful things and it gets implemented and then it gets pushed to the back.” In many sites, child welfare professionals tied such skepticism to an overall lack of knowledge about, and experience with, EBIs among staff, who were accustomed to selecting parts of programs they believed would work best for families. One supervisor clarified, “What happens is the staff are given a list of curricula that they can utilize that they’ve all been trained on, and so truly it’s not one specific [program from start to finish].” A provider in the control condition commented, “I don’t know if there’s actual curriculum that we can cite... we are kind of fly-by-the-seat-of-our-pants and address issues as they arise.” Many child welfare professionals reported unfavorable perceptions of EBIs in general as rigid and overly structured.

As these comments indicate, many child welfare professionals perceived SafeCare as out of step with their values regarding how to provide services to families. Providers commonly asserted that SafeCare was too scripted, making them feel like their interactions with families were not genuine. A frequently echoed statement from providers was, “We just go with what the child is needing and what the family needs.” Many supervisors and providers also maintained that SafeCare was difficult to implement because the primary needs of the family (e.g., adequate shelter, medical concerns, and food insecurity) were not being met. They also reported trouble finding families who were both eligible for SafeCare and willing to remain involved with services for the length of time required to complete the program. Ultimately, administrators at low implementation sites recognized that more preparation to incorporate SafeCare into the existing work of their organizations would have prevented many of these problems. One noted, “No one questioned the merits of the model. Everyone questioned the compatibility.”

In contrast, while child welfare professionals in high implementation sites also recalled concerns about SafeCare’s rigidity and required time commitment in the early Implementation phase, they stated that these concerns had diminished over time. In fact, these professionals portrayed SafeCare’s structure as reflective of its quality. Similarly, the supervisors and providers at this site portrayed requirements to adhere to the SafeCare model as beneficial, rather than as an additional burden, to both families and sites delivering services, contributing to a documentable graduation rate that made it easier to measure progress.

#### Requirements of RCT participation at the provider level

Mirroring the problems participants reported with lack of fit and poor preparation pertaining to SafeCare, it was also clear that participation in the RCT itself suffered from a lack of planning to integrate the requirements of the RCT into the workloads of child welfare professionals, leading to confusion around roles, responsibilities, and tasks. Throughout implementation, staff at multiple sites commonly experienced difficulties securing referrals for SafeCare to meet the goals of the RCT. A supervisor commented on this pervasive problem, “We have not had a ton of referrals that met the criteria for SafeCare one way or the other so that’s been a big challenge.” An administrator at another site mused, “The difficulty for the staff was, ‘If I’m seeing a mother with a newborn child who also has a four-year-old, which [child] gets the SafeCare intervention? Which becomes the SafeCare case?’” Because receipt of SafeCare was voluntary at some sites, it was challenging for providers to recruit parents for the RCT when that recruitment involved getting an additional service. Some providers reportedly did not bother offering SafeCare to clients because they assumed they would not be interested, while others expressed discomfort at being asked to recruit parents. One explained, “I felt like I’m not a good sales person... I’m sure I didn’t deliver the message as well as the project would like me to.... [I]t just felt a little uncomfortable for me.” Some professionals contended that the requirement to help with recruitment reflected a lack of understanding of their burdensome workload, commenting for example, “I don’t feel like... [the research team members]... know our job and what we’re actually dealing with all the time so it’s really hard for them to get a whole grasp of the barriers or trying to implement it.”

Even after parents were successfully recruited and consented, the requirements of the RCT continued to pose problems for some providers. In at least one site, providers complained that the local data collectors who were hired to conduct assessments did not complete their tasks in a timely manner, explaining, “[The data collectors have] not been very timely with setting up baselines with our families so that’s turned a lot of our families off. We’ve had families back out of wanting to be part of the study.” Another provider suggested that such issues were a result of naivety on the part of the researchers, commenting, “I don’t know if [the researchers] realized [that]... a lot of our parents are very hard to get ahold of and that’s been a struggle with even getting the [study] started."

Another common issue related to the RCT was trouble with the smartphone app that frontline staff were asked to use to record information and conduct surveys. In some cases, the app did not function correctly, staff were not trained sufficiently in its usage, or the app was not compatible with the technology that staff were already using. Together, these problems reportedly contributed to feelings of resentment among providers toward the study and their superiors. In one exemplary comment, a provider stated, “There’s no sympathy on our part [from superiors and researchers] to do your homework. ‘Just do it. Do it.’”

Finally, participants opined that the study requirement to keep some staff in a control condition led to a lack of investment that undermined the RCT. Providers in the control condition were aware that they had to comply with the requirements of the RCT (i.e., recruiting families and using the smartphone app to document sessions) without yet receiving many of the benefits of participating (e.g., coaching). One administrator recounted having to bring in a SafeCare coach to explain the study more thoroughly to the control providers to motivate them to recruit more clients. Ultimately, an administrator observed that the challenges staff experienced complying with research requirements ultimately undermined their buy-in for SafeCare, explaining, “Because implementation has been challenging, then all of the struggles of the research, and all of the struggles of implementation are now attached to the model.”

## Discussion

This study focuses on the experiences of child welfare professionals involved in a largescale trial of SafeCare, a structured behavioral parenting program, to illuminate factors in the implementation environment that facilitated or impeded success in the adoption of SafeCare. Our findings indicate that in high implementation sites, where SafeCare was successfully instantiated, child welfare professionals reported positive experiences with SafeCare and positive effects on families. While providers in these sites reported that they had initially been concerned about increased workloads, lack of flexibility in service delivery, and the feasibility and fit of maintaining the intervention within their organizations, they explained that these concerns diminished over time. In contrast, this positive change did not occur in low implementation sites where staff struggled to integrate SafeCare into their work. In these sites, providers reported persistent negative perceptions of the program. Our findings suggest that concerns related to the intervention’s “fit” with organizational values or its “burden” on providers or parents resulted from a variety of system- and organization- level barriers that adversely impacted implementation in these sites. Notably, a lack of integration of SafeCare into child welfare contracts, referral processes, and organizational procedures made it difficult for both sites and their frontline staff to integrate the new intervention into their existing work routines and caseloads [23]. Further, many of the barriers adversely affecting these sites appeared to be out of the control of the implementing organizations. These included turnover among organizational leaders, as well the burdens associated with implementation of the research study itself.

### Enhancing implementation of new interventions

While providers in the high implementation sites sometimes struggled with the tasks associated with the RCT, they were generally able to accommodate the responsibilities of the RCT and contribute to the success of their organizations in implementing SafeCare. Consequently, study findings indicate several methods to enhance implementation through establishing bridging factors between inner and outer contexts and decrease the burden of participating in an RCT [23]. Beginning in the Exploration and Preparation stages, enthusiastic planning and alignment of consistent support from system and organizational leaders garnered buy-in at every step of the implementation process—findings in keeping with the substantial body of research highlighting the importance of knowledgeable, supportive, perseverant, and proactive leadership in creating the organizational climate to implement EBIs [24–26].

In the Preparation and Implementation phases, ready uptake of SafeCare was aided by perceptions that EBIs would help families, increase the competitiveness of organizations, and contribute to existing organizational cultures of “trying new things.” Study findings indicate additional ways to mobilize leadership throughout the Implementation and Sustainment phases to enhance staff buy-in to new innovations. First, continuity of leadership support over time was vital to the success of some sites. Second, it is crucial to ensure the alignment of leadership across levels [27]. In high sites, communication among administrators, supervisors, and providers represented a crucial bridging factor between outer and inner contexts that enabled leaders to make decisions based on knowledge of clients’ needs and providers’ workloads, while frontline staff felt empowered to give feedback [23, 28]. Third, as providers are frequently concerned with what they perceive as the inflexibility of SafeCare, leaders should emphasize coaching support to help staff develop skills in tailoring SafeCare to family needs [29].

In addition to amplifying facilitating factors, the Preparation phase offers key opportunities to reduce barriers that impede the implementation of SafeCare and compliance with an RCT design. Stakeholders must carefully consider and demarcate the allocation of tasks and requirements related to the provision of SafeCare versus the RCT before implementation [30]. It is important to be clear-eyed about the actual time and effort involved for providers in the provision of SafeCare in the context of an RCT. When new models like SafeCare are added to existing service packages, it may also be important to consider de-implementing ineffective services to accommodate providers’ workloads and avoid overwhelming families [31]. Finally, the referral process by which families enter the program should also be clearly established in advance to remove uncertainty around whether families should receive SafeCare and which families are eligible for the RCT.

### Enhancing participation in research

Although the status of the RCT as the “gold standard” of research evidence is increasingly being challenged, RCTs still often drive funding and policies related to how interventions are selected and provided [32]. However, this research illuminates the critical ways in which participating in an RCT may influence providers’ experiences of implementing a new EBI and, in some cases, even contribute to implementation failure. In this study, involvement in an RCT burdened staff with new responsibilities, such as recruiting families and coordinating services with the timing of research assessments, for which they were not prepared and sometimes did not understand the necessity. These were compounded by other additional tasks, such as using an unfamiliar app. Such responsibilities appeared especially superfluous for providers in the control condition who were not implementing the new model and may not have understood their role in the research. Struggles with research-related tasks contributed to sentiments among frontline staff that the researchers were out-of-touch with the realities of their work and the needs of the families they served. These experiences raise the possibility that frustration and resentment stemming from the RCT design may poison the waters for generating evidence for promising interventions.

As frontline staff bear the brunt of service provision and participation in studies like this one, researchers should also work to alleviate the burdens of study involvement and promote buy-in among frontline staff not only for the program but also for the research itself. A key strategy is to use participatory research methods to cultivate enthusiasm and solicit feedback from frontline staff as partners in the RCT [33]. Beginning in the RCT design phase, researchers and organizational administrators should initiate ongoing planning discussions with staff on eligibility criteria, enrollment processes, RCT instrumentation, and other issues based on their experience in the field. Although such discussions can be challenging to accommodate given turnover among frontline staff and the extended timeline involved in designing, funding, and implementing a trial, they allow service providers to become partners and coproducers of knowledge in the research process. A complementary approach is described in scholarship on knowledge exchange, which underscores the importance of replacing a one-way model of knowledge transfer from researchers to other stakeholders with an interdependent model, in which all stakeholders have experiential knowledge to share in the design and conduct of research [34]. This approach also requires more extensive reflection on the part of researchers about potential barriers to effective knowledge exchange and methods to overcome them.

Secondly, researchers must separate the demands of providing an EBI from the tasks associated with an RCT protocol both conceptually and pragmatically. Researchers should communicate clearly about study-related tasks so that staff know what is permanent and what is temporary. If possible, researchers should avoid tasking frontline staff with recruitment, randomization, and consent responsibilities, and should streamline RCT procedures, focusing on those that all stakeholders agree are essential.

### Limitations

Our use of the SIC allows us to identify common characteristics of high and low implementation environments; however, we cannot fully capture the nuanced variations among sites, nor can we completely disentangle influences on SafeCare implementation related to study participation from those that stemmed from other aspects of the implementation environment. Future research should examine this distinction by building in mechanisms for reflexivity about study participation on the part of both researchers and participants [35, 36]. This study centered the voices of child welfare professionals participating in this research. To maintain our focus on the implementation environment, we did not include the perspectives of parents and caregivers who received the intervention. Their experiences with the SafeCare intervention will be the focus of a future analysis.

## Conclusion

As this research confirms, the implementation of EBIs like SafeCare involves complex processes that are subject to a wide variety of inner- and outer-context influences, including effective organizational and system leadership, proactive planning, and alignment of enthusiasm from administrators to frontline service providers. Our findings also provoke a question: how much has research in this field been impacted by joint efforts to implement a new practice and study it at the same time? Successful adopters of new interventions are likely to be agencies and organizations with the existing capacity for implementation and the infrastructure to support the requirements of a research project. However, it remains difficult to assess how much unsuccessful implementation efforts may have been hindered by the layering of research requirements on top of implementation. The next generation of methods in implementation science must include approaches that are designed to be less burdensome on the already over-worked frontline providers of services like child welfare, while still answering scientific questions about how to successfully implement EBIs.

## Data Availability

The data used and analyzed during the current study are available from the corresponding author on reasonable request and permission of the Principal Investigator.

## Abbreviations

CBO: Community-based organization
EBI: Evidence based intervention
EPIS: Exploration, Preparation, Implementation, and Sustainment model
NSTRC: National Safecare Training and Research Center
RCT: Randomized controlled trials
SIC: Stages of Implementation Completion

## Footnotes

1 To protect the anonymity of research participants, we use “they” as the singular third-person pronoun throughout this manuscript, in keeping with recommendations in the 7^th^ edition of the *Publication Manual of the American Psychological Association.*
